# Cell type-specific gene expression patterns associated with posttraumatic stress disorder in World Trade Center responders

**DOI:** 10.1038/s41398-018-0355-8

**Published:** 2019-01-15

**Authors:** Pei-Fen Kuan, Xiaohua Yang, Sean Clouston, Xu Ren, Roman Kotov, Monika Waszczuk, Prashant K. Singh, Sean T. Glenn, Eduardo Cortes Gomez, Jianmin Wang, Evelyn Bromet, Benjamin J. Luft

**Affiliations:** 1 Department of Applied Mathematics and Statistics, Stony Brook University, Stony Brook, NY USA; 2 Department of Medicine, Stony Brook University, Stony Brook, NY USA; 3 Department of Family and Preventive Medicine, Stony Book University, Stony Brook, NY USA; 4 Department of Psychiatry, Stony Book University, Stony Brook, NY USA; 5 Center for Personalized Medicine, Roswell Park Comprehensive Cancer Center, Buffalo, NY USA; 6 Department of Biostatistics and Bioinformatics, Roswell Park Comprehensive Cancer Center, Buffalo, NY USA

## Abstract

Posttraumatic stress disorder (PTSD), a chronic disorder resulting from severe trauma, has been linked to immunologic dysregulation. Gene expression profiling has emerged as a promising tool for understanding the pathophysiology of PTSD. However, to date, all but one gene expression study was based on whole blood or unsorted peripheral blood mononuclear cell (PBMC), a complex tissue consisting of several populations of cells. The objective of this study was to utilize RNA sequencing to simultaneously profile the gene expression of four immune cell subpopulations (CD4T, CD8T, B cells, and monocytes) in 39 World Trade Center responders (20 with and 19 without PTSD) to determine which immune subsets play a role in the transcriptomic changes found in whole blood. Transcriptome-wide analyses identified cell-specific and shared differentially expressed genes across the four cell types. *FKBP5 and PI4KAP1* genes were consistently upregulated across all cell types. Notably, *REST* and *SEPT4*, genes linked to neurodegeneration, were among the top differentially expressed genes in monocytes. Pathway analyses identified differentially expressed gene sets involved in mast cell activation and regulation in CD4T, interferon-beta production in CD8T, and neutrophil-related gene sets in monocytes. These findings suggest that gene expression indicative of immune dysregulation is common across several immune cell populations in PTSD. Furthermore, given notable differences between cell subpopulations in gene expression associated with PTSD, the results also indicate that it may be valuable to analyze different cell populations separately. Monocytes may constitute a key cell type to target in research on gene expression profile of PTSD.

## Introduction

Posttraumatic stress disorder (PTSD) is a complex disorder that affects ~7% of the US population^1^. PTSD develops in response to exposure to traumatic events and is characterized by emotional numbing, intrusive memories, avoidance, and hyperarousal^2^. PTSD can lead to cognitive, social and occupational impairment, and is associated with neurodegeneration^3,4^ thus causing substantial social and economic burden for individuals affected by this condition^5^. The etiology of PTSD is not well understood. However, genetic studies involving twin, candidate gene, and genome-wide association analyses^6^ have revealed that heritability of PTSD is moderate in size (ranging from 30 to 40%)^7,8^, and that genetic factors may play an important role in the vulnerability to, and resilience following, trauma exposure in PTSD^9^. PTSD is also consistently associated with altered functioning of the immune system, including increased levels of circulating C-reactive protein and pro- and anti-inflammatory cytokines^10–12^. To date, the link between genetic and immunologic processes in PTSD remains unclear.

Gene expression analyses can identify critical downstream biological process associated with genetic and epigenetic variations and thus can potentially inform efforts to identify biomarkers for PTSD^13^. Transcriptome-wide gene expression profiling has emerged as the preferred approach for explicating gene regulation, because it allows for an unbiased investigation of expression patterns without *a priori* knowledge of genetic risk factors^14^. Taking such hypothesis-free approach, previous studies, including work from our group, identified differentially expressed pathways related to immune functions and inflammation to be most prominent in PTSD^15–17^.

As direct sampling of the brain is not feasible, most prior studies were performed using whole blood or unsorted peripheral blood mononuclear cell (PBMC)^13,15–22^. To a large extent, gene expression patterns in blood are consistent with patterns observed in brain^23,24^, suggesting that the molecular signature of PTSD may be obtained outside the brain. An additional strength of focusing on blood tissue is its feasibility as a potential clinical biomarker. However, blood is a complex tissue that consists of several populations of cells, and each has a distinct gene expression profile. Alterations in immune regulatory networks are expected to have functional consequences primarily in certain subsets of immune cells. Thus, analyses of whole blood are likely to weaken the signal. Indeed, in autoimmune diseases, analyses of gene expression in specific cell types reveal stronger links to target disease than analyses of whole blood^25–27^. As PTSD is implicated in immune responses and suggestive evidence links it to autoimmune diseases^28–31^, studying genetic alterations in isolated immune cell subsets may provide a clearer understanding of the link between PTSD and immune function. Noting this, one previous case-control study examined gene expression in isolated monocytes in 49 men with and without PTSD^32^, and identified three significantly downregulated genes (*PF4*, *SDPR*, and *HIST1H2AC*) despite finding no evidence of chronic inflammation. The authors also found a large range of genes with clinically meaningful effect sizes that were predominantly under-expressed in PTSD, suggesting decreased gene activation in immune cells.

To our knowledge, no study of PTSD to date has profiled gene expression in isolated immune subsets other than monocytes. The current study addressed this gap by conducting a transcriptome-wide gene expression study using the state-of-the-art RNA sequencing (RNA-Seq) approach on RNA derived from the main subsets of isolated immune cells retrieved from peripheral blood. To this end, we analyzed blood from 39 male responders to the WTC disaster with and without WTC-connected PTSD whose whole-blood transcriptome had previously been studied^15^. We compared gene expression profiles within four isolated subsets of cells: CD3+CD4+ T cells (CD4T), CD3+CD8+ T cells (CD8T), CD19+ B cells (B cells) and CD14+ monocytes (monocytes) to determine the immune subsets that were responsible for the changes in the transcriptome found in our previous study^15^. Also, we performed candidate gene analyses to test replicability of the only previous study of PTSD gene expression in monocytes, which identified downregulation of *PF4*, *SDPR*, and *HIST1H2AC* in this cell type. To better understand genetic predisposition to PTSD, biological pathway analyses were performed on differentially expressed genes. The results from isolated immune cells were compared to results obtained from PBMC and whole blood. This study tested the hypothesis that both common and distinct gene expression patterns in PTSD would be observed in immune cells relative to PBMC and whole blood.

## Methods

### Setting

The World Trade Center (WTC) disaster was a catastrophic event that simultaneously exposed tens of thousands of individuals, including WTC responders who worked on the site during rescue and recovery operations, to acute psychological and physical trauma^33,34^. Stony Brook University manages the second largest program and monitors responders residing on Long Island, NY^35^. Most responders in the Stony Brook program are male, worked as police, and were aged 39 in September 2001. We previously found that 18% of the cohort developed WTC-connected PTSD, and 10% had chronic WTC-PTSD^34^.

### Participants and clinical assessment

This study utilized blood samples from a subset of participants (*n* = 39) based on their polygenic expression scores from a previous study in which we had already demonstrated significant gene dysregulation^15^. Details on recruitment, clinical assessment, and biomarker sampling were described previously^15^. The 39 participants in the present study were non-smoking males (mean age = 51.4, SD = 8.94); 20 had a history of DSM-IV WTC-PTSD determined using the Structured Clinical Interview for DSM-IV^36^ and had highest polygenic expression scores, while 19 did not and had lowest polygenic expression scores. Interviews were conducted concurrently to blood draw by professional interviewers under supervision of clinical psychologists.

### Peripheral blood mononuclear cell (PBMC) isolation and freezing

Whole-blood samples were collected in BD Vacutainer CPT cell preparation tubes containing sodium heparin as the anti-coagulant (BD, Franklin Lakes, NJ, USA). The CPT tubes were processed within 2 h on at room temperature according to the manufacturer’s instruction. The purified PBMC cells were aliquoted in 90% FBS + 10% dimethylsulphoxide (DMSO) with cell concentration at >2 × 10^7^ cells/ml per cryovial (0.75 ml per cryovial). To ensure stepwise temperature decrease^37^, the cryovial were placed in Mr Frosty’ containers (Nalgene/Thermo Fisher, Rochester, NY, USA) with isopropyl alcohol medium in −80 °C freezers for 24–48 h. The cryovials were transferred quickly into liquid nitrogen for long term storage. The crypreserved PBMC samples were shipped with a dry-ice package to the Roswell Park Cancer Center and stored at −8 °C for flow sort sub-cell separation and RNA extraction within 1–2 weeks.

### Flow sorts on frozen PBMC, total RNA isolation, library preparation, and sequencing

Frozen PBMC were thawed, restored, washed, and counted. An aliquot of cells was resuspended in PBS containing 0.1% BSA (PBS/BSA), blocked with mouse IgG, and labeled at room temperature with the mouse anti-human antibodies. The cells were then washed in PBS/BSA, resuspended in 1 ml PBS/BSA, filtered through a 35 micron mesh, and diluted with 1 ml sort buffer. Four-way sorting was performed on a BD FACSAria II sorter, where monocytes, B cells, CD4T, and CD8T cells were sorted simultaneously. The purification of total and small RNA was prepared using the miRNeasy mini kit (Qiagen). Quantitative assessment of the purified total RNA was accomplished using a Qubit Broad Range RNA kit (Thermo Fisher), which was then qualitatively evaluated by a 2100 Bioanalyzer (Agilent technologies). The sequencing libraries were prepared with the TruSeq Stranded Total RNA kit (Illumina Inc) and sequenced on a HiSeq2500 sequencer using a 100 cycle single-read cluster kit. Additional details are provided in Supplementary Materials.

### RNA-Seq data preprocessing

Alignment was performed using the TopHat2 software^38^ which utilizes Bowtie2^39^ (http://bowtie-bio.sourceforge.net/bowtie2/index.shtml) on RefSeq (NCBI Reference Sequence Database) annotation and human reference genome (GrCh37-hg19 version)^40^. Spliced alignment of the reads to the reference genome was done with the TopHat2 software allowing a maximum of one mismatch per read; quality control was done using RSeQC. Other genomic related data were obtained using UCSC’s genome repository.^41^ Quality control for raw reads was performed with FastQC,^42^ and adapter trimming was done with cutadapt^43^. The number of read counts mapping to each gene was computed using htseq-count.^44^

### Estimation of batch effects

The potential for batch effects was estimated from the log-normalized gene counts data using surrogate variable analysis approach for sequencing data (svaseq)^45^. The estimated surrogate variables were included in differential expression analyses as adjustment factors. Proportions of CD4T, CD8T, monocytes and B cells estimated from cell sorting were included in differential expression analysis for PBMC and whole blood as adjustment factors. We also compared the estimated cell subsets from two computational tools, namely CIBERSORT^46^ and xCell^47^ software. CIBERSORT was developed based on deconvolution method using microarray datasets, whereas xCell was based on integration of gene set enrichment analysis and deconvolution method on both RNA-Seq and microarray datasets. The estimated cell type abundances between PTSD and non-PTSD were compared using the two sample *t*-tests.

### Differential expression analysis

Differential expression analyses of RNA-Seq data generated from isolated CD4T, CD8T, B cells, and monocytes was performed using DESeq2^48^ software based on multivariable-negative binomial generalized linear models that adjusted for age, race, and estimated surrogate variables to account for potential batch effects. Genes with low expression were filtered using the cpm (count-per-million) function in edgeR^49^. A total of 15,947 genes were included in the analysis after filtering. Statistical significance was assessed via the Wald test using appropriate contrasts to identify differentially expressed genes associated with PTSD for each cell type. A false discovery rate (FDR)^50^ control was used to account for multiple testing. FDR < 0.05 was used to identify statistically significant genes. Post hoc analysis comparing the number of differentially expressed genes at a range of nominal *p*-value thresholds was conducted to evaluate the signal strength of differential expression associated with PTSD across cell types. A joint analysis, adjusting for cell types and the confounders described above was also conducted to identify differentially expressed genes associated with PTSD. Heatmaps and volcano plots were used to visualize gene expression patterns. Principal component analysis (PCA) was performed on the matrix of normalized gene counts. The first three principal components (PC1, PC2, and PC3) were used to visualize global gene expression patterns. Pearson correlation coefficients were computed on the estimated log2 fold change from DESeq2 in order to conduct post hoc comparisons of similarities and differences across cell types.

### Candidate gene analysis

The association between PTSD and gene expression was examined for previously implicated genes in monocytes (*PF4, SDPR, HIST1H2AC*)^32^. The estimated log2 fold change, nominal and Bonferroni adjusted *p*-values for these three genes were reported.

### Pathway and gene ontology analyses

Pathway and gene ontology analyses were carried out using the over-representation via the Bioconductor package clusterProfiler^51^. Over-representation analysis was performed on the top 100 genes ranked by *p*-values from the differential expression analysis using the function enrichGO. In total, 5414 gene ontologies, including biological processes, molecular functions, and cellular components (the range of genes per gene set was 15–500) were tested. Statistically significant gene sets were corresponded to those with FDR < 0.05 from over-representation analyses.

### Discriminant analysis via elastic net regularized regression

To evaluate the synergistic effect of multiple genes in discriminating PTSD status within each cell type, we randomly divided the 39 samples into 29 training and 10 test samples. The objective of this analysis is to provide another analytic framework to compare the signal-to-noise ratio of the different cell types. Within the training set, the elastic net^52^ algorithm with threefold cross-validation was applied to the top 1000 genes identified from the differential expression analyses of the current training set to ensure unbiased selection of candidate features. The area under receiver operating curve (AUC), based on the model obtained from the training set, was computed on the test set. The random partitioning of training and test samples was repeated ten times, and the average AUC of the test set was reported.

Additional statistical analyses including weighted gene co-expression network analysis^53^ and differential expression analysis comparing each cell type pair stratified by PTSD status are provided in Supplementary Materials. An overview of the cell-specific RNA-Seq data analysis pipeline was given in Supplementary Figure 1.

### Code availability

Script for the main analysis is available at http://www.ams.sunysb.edu/~pfkuan/CellGeneExp.

## Results

### Participant characteristics

We did not find significant group difference on the exposure severity between cases and controls. Furthermore, we compared controls and PTSD cases on hallmark WTC-related disorders: lower respiratory symptoms (LRS) and gastroesophageal reflux disease (GERD) symptoms, and did not find a group difference. Finally, the two groups did not differ on BMI, and demographic variables (Table 1).

**Table 1:** Clinical characteristics of the 39 samples

All	Case *N* = 20	Control *N* = 19	*P*-value
*Age*			
Mean (SD)	53.35 (8.12)	49.37 (9.51)	0.169
Race *N* (%)			
Caucasian	17 (85)	19 (100)	0.231
Other	3 (15)	0 (0)	
*PCL*			
Mean (SD)	56.00 (8.43)	18.01 (1.54)	<0.01
*Polygenic score*			
Mean (SD)	0.46 (0.18)	0.30 (0.12)	<0.01
*BMI*			
Mean (SD)	31.90 (5.31)	32.06 (7.32)	0.937
*LRS N (%)*			
Yes	14 (70)	8 (42)	0.111
No	6 (30)	11 (58)	
*Exposure*			
Mean (SD)	2.06 (0.97)	1.60 (0.98)	0.195
*GERD N (%)*			
Yes	17 (85)	11 (58)	0.082
No	3 (15)	8 (42)	

The *p*-values were computed from *t*-test (for continuous variables) and Fisher’s exact test (for categorical variables). The polygenic scores were computed based on our previous paper on whole blood^3^

### Differentially expressed genes across different cell types

**Fig. 1:**
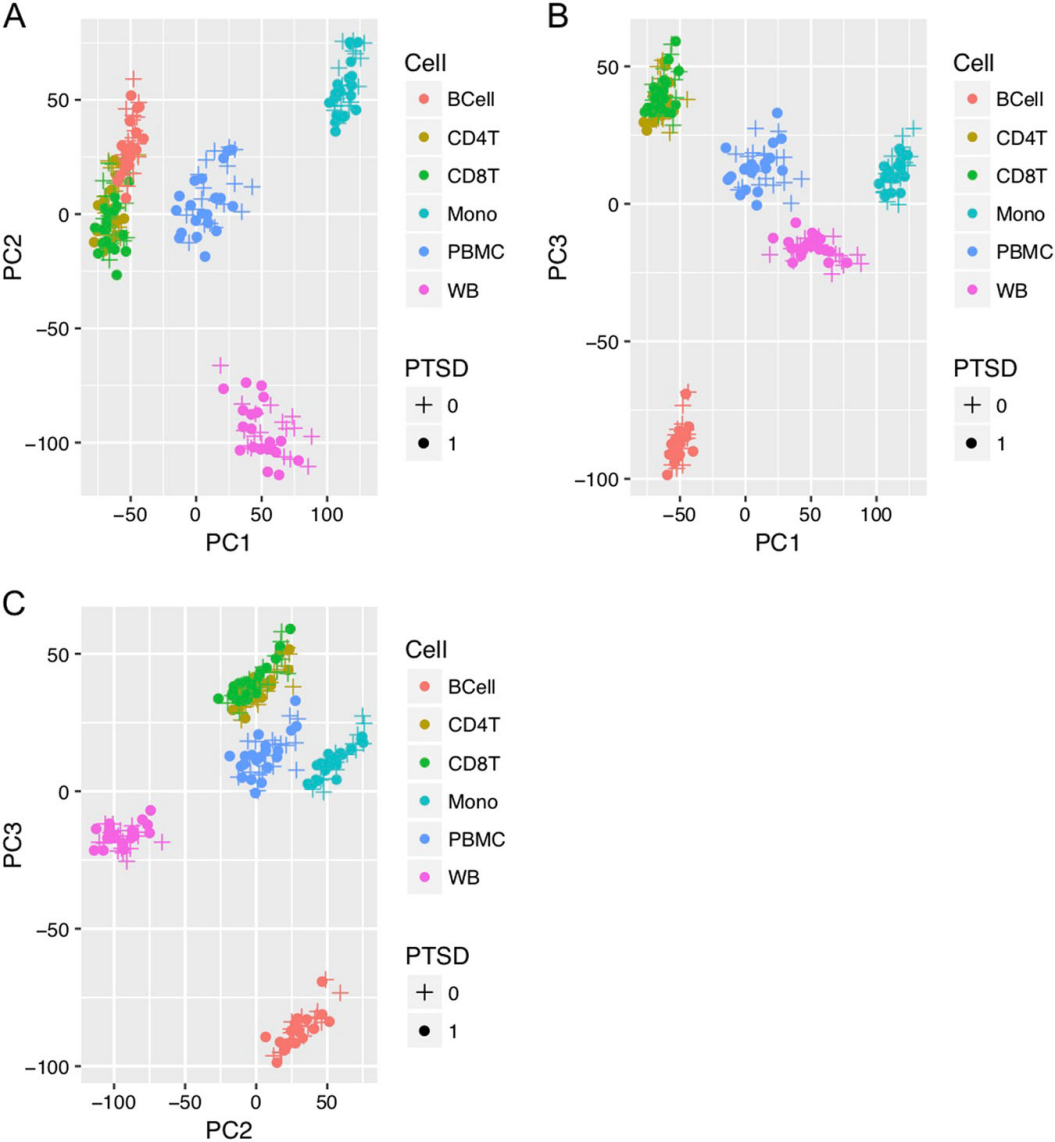
Principal component analysis (PCA) of transcriptome-wide normalized gene expression counts assessing the difference in immune cell types. Each dot represented a sample, color coded by cell type (Red: B cells, Sage: CD4T, Green: CD8T, Turquoise: monocytes, Sky blue: PBMC, purple: whole blood, plotting symbol+: control, solid circle: PTSD). **a** *x*-axis denotes the value of PC1, *y*-axis denotes the value of PC2. **b** *x*-axis denoted the value of PC1, *y*-axis denotes the value of PC3. **c** *x*-axis denoted the value of PC2, *y*-axis denotes the value of PC3

**Fig. 2:**
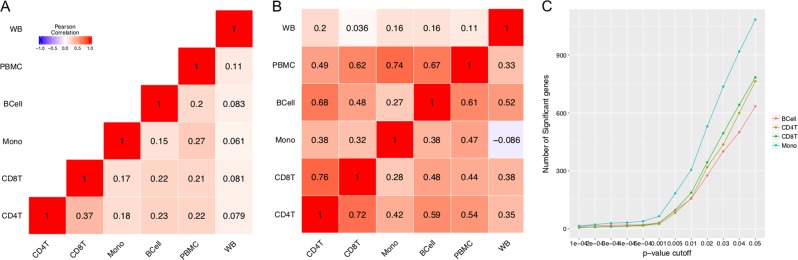
Differential expression analysis. **a** Pairwise correlation coefficients between cell types comparing the estimated log2 fold change across all genes from DESeq2 for the differential expression analysis in PTSD. **b** Pairwise correlation coefficients of the estimated log2 fold change for the top 100 genes from DESeq2 for the differential expression analysis in PTSD for each cell type (row). The matrix is asymmetric because each row corresponds to different gene list, e.g., rows 1 is based on the top 100 differentially expressed (DE) genes in whole blood, whereas row 2 is based on top 100 DE genes in unsorted PBMC. **c** Number of differentially expressed genes in PTSD (*y*-axis) at different nominal *p*-value thresholds (*x*-axis) for each cell type

Principal component analysis (PCA) of transcriptome-wide normalized gene expression counts (Fig. 1a–c) showed significant cell type differences, with CD4T and CD8T showing the largest degree of similarity. The PCA plot also showed that most of the gene expression changes observed were related to the specific cell types instead of PTSD status. Similar patterns were also observed when we restricted the comparison to the top 1000 most variable genes across the different cell types (Supplementary Figure 4A) or within each cell type (Supplementary Figure 4B-E). As expected, gene expression of unsorted PBMC fall in between that of the cell types. Transcriptome-wide estimated log2 fold change from DESeq2 for the differential expression analysis comparing WTC-PTSD to unaffected responders also showed that CD4T and CD8T were most similar to each other (*r* = 0.37, *p* < 0.001) followed by B cells, whereas monocytes and whole blood were least similar (*r* = 0.061, *p* < 0.001) (Fig. 2a). Correlations between differential gene expression profiles in whole blood and in immune cell types ranged between 0.06 and 0.11. The top 100 genes identified within CD4T showed correlations ranging from 0.35 to 0.72 with other cell types. The top 100 genes identified from unsorted PMBC correlations ranging from 0.49 to 0.74 with the isolated immune subsets and 0.33 with whole blood. The top 100 genes identified from whole blood showed correlations ranging from 0.036 to 0.2 with other cell types (Fig. 2b). These results suggested that the transcriptome-wide differential gene expression observed in whole blood was partially attributed to differential gene expression in granulocytes that were removed during the separation of PBMC from whole blood.

The proportion of CD4T estimated from cell sorting was different between case and control (*p*-value 0.019, Supplementary Figure 3A). Similar pattern of elevated CD4T abundances in PTSD was also observed using the cell subtypes estimates from CIBERSORT and xCell, although they did not meet statistical significance (Supplementary Figure 3B, 3C). The correlations between the cell subtype estimates from CIBERSORT and cell sorting were >0.75 for all cell types except CD8T (Supplementary Figure 3D); whereas, the correlations between xCell and cell sorting were lower in these cell types, except for B cells. In addition, the estimated log2 fold change and the negative log *p*-values from the differential expression analysis comparing PTSD to control, adjusting for cell heterogeneity using cell sorting proportions exhibit a higher correlation with CIBERSORT for both the unsorted PBMC and whole-blood analysis, compared to the adjustment using xCell (Supplementary Figure 8). CIBERSORT outputs the estimated proportions, which were directly comparable to the output of cell sorting. On the other hand, xCell outputs enrichment scores for the cell types, which may not be interpreted as proportions.

**Fig. 3:**
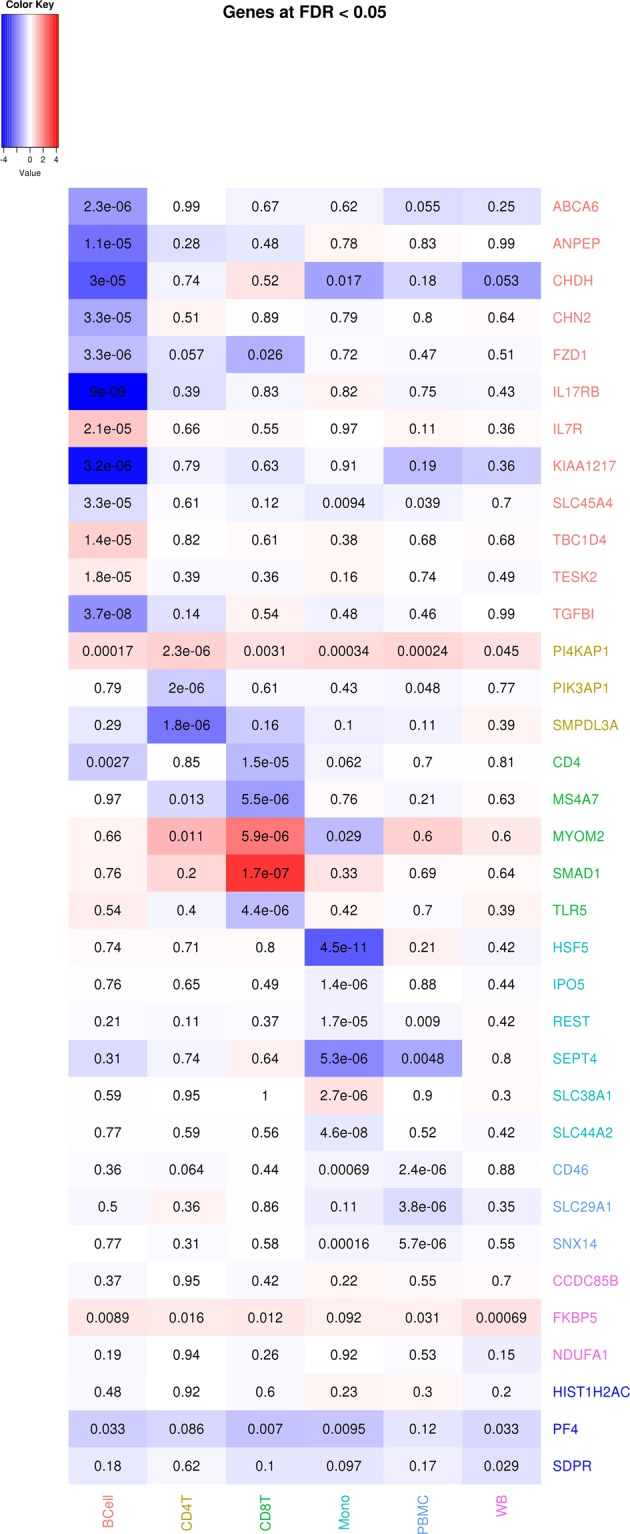
Heatmap of the differentially expressed genes at FDR < 0.05. The gene names were color coded according to the cell type in which they were found to differentially expressed (Red: B cells, Sage: CD4T, Green: CD8T, Turquoise: monocytes, Sky blue: PBMC, purple: whole blood). The last three genes in dark blue color corresponded to the differentially expressed genes identified by Neylan et al.^32^. The number printed in each heatmap cell corresponded to the estimated unadjusted *p*-value from DESeq2, and the color corresponded to the magnitude of the estimated log2 fold change (blue: downregulated in PTSD, red: upregulated in PTSD)

Volcano plots (Supplementary Figure 5A-D) depicting global differential gene expression patterns indicated an approximately equal amount of over- and under-expression comparing WTC-PTSD to non-PTSD across the four cell types. At FDR < 0.05, 3, 5, 6, 12, and 3 genes were identified to be differentially expressed in CD4T, CD8T, monocytes, B cells, and unsorted PBMC, respectively (Fig. 3). Across different nominal *p*-value thresholds, monocytes identified the largest number of differentially expressed genes among the immune cell subsets (Fig. 2c), suggesting that monocytes contained the strongest gene expression differences in PTSD compared to CD4T, CD8T, and B cells.

Most of the genes identified to be statistically significant at FDR < 0.05 were specific to each cell type (Fig. 3), except for *PI4KAP1* which was upregulated in CD4T (FDR < 0.05) and the rest of the cell types with nominal *p*-values < 0.05. In our previous study^15^, *FKBP5* was found to be upregulated in whole blood in a sample of 282 responders. This gene remained consistently upregulated in this subset of 39 responders across all cell types (nominal *p*-values <0.05 in CD4T, CD8T, B cells, unsorted PBMC and whole blood, and nominal *p*-value <0.1 in monocytes). On the other hand, *NDUFA1* and *CCDC85B* were previously found to be downregulated in whole blood. *NDUFA1* exhibited weak downregulation effect sizes in all cell types (nominal *p*-values >0.05), whereas *CCDC85B* did not reach statistical significance in this subset of 39 responders. The proportion of downregulated genes in monocytes vary between 40 and50% across different nominal *p*-value thresholds (Supplementary Figure 6).

The joint differential expression analysis across the four cell subsets for PTSD status identified 34 genes at FDR < 0.05 (Supplementary Figure 7). Among these 34 genes, only *PI4KAP1* was in common with the cell-specific gene expression analysis of CD4T. *FKBP5* was among these 34 genes and was upregulated in the joint analysis, consistent with our previous findings.

### Candidate gene analysis

As noted above, *PF4, SDPR, HIST1H2AC* were previously identified to be downregulated in monocytes in male subjects with PTSD using microarrays^32^. Among these three genes, *PF4* (*p* = 0.01, Bonferroni *p* = 0.029) was also found to be at least marginally downregulated in monocytes in this study. Results also extended to other cell types and whole blood (Fig. 3). *PF4* was also significantly downregulated (FDR < 0.05) in the joint differential expression analysis of the four cell types for PTSD status (Supplementary Figure 7).

### Pathway and gene ontology analyses

**Fig. 4:**
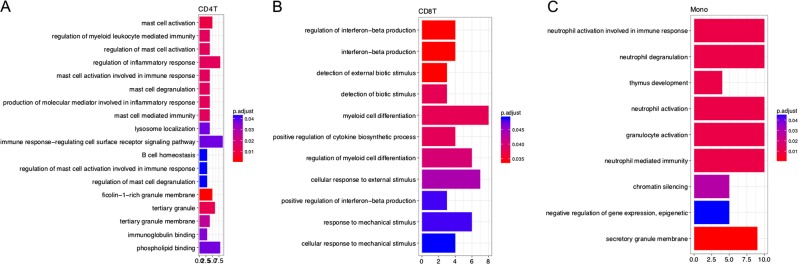
Statistically significant gene ontologies at FDR < 0.05 among the top 100 genes identified to be differentially expressed in PTSD for each cell type. *x*-axis denoted the number of differentially expressed genes overlapping with each gene ontology. No gene set was significant at FDR < 0.05 for B cells

Pathway and gene ontology analyses identified 18, 11, and 9 gene sets to be enriched at FDR < 0.05 among the top 100 genes associated with PTSD in CD4T, CD8T, and monocytes, respectively (Fig. 4). No gene set was significant at FDR < 0.05 for B cells. Most of the enriched gene sets were related to immune responses and inflammation. Gene sets related to mast cell activation and regulation emerged as the top ontology for CD4T. Gene sets related to interferon-beta production were identified as the top ontology for CD8T, whereas neutrophil-related gene sets were the top ontology for monocytes.

### Cell-specific discriminant analysis for PTSD

The average AUC values over the 10 random splits for CD4T and monocytes were 0.666 and 0.735, respectively; whereas, the average AUC values for CD8T and B cells were ~0.5 (equivalent to random guess). Despite the small sample size, monocytes showed promising discriminant power in differentiating PTSD from healthy controls. This result was also consistent with the earlier finding that at a fixed *p*-value threshold, a higher proportion of differentially expressed gene in PTSD was detected in monocytes compared to other immune cell types.

## Discussion

In order to enhance our understanding of gene regulation in PTSD, the current study was the first to characterize the gene expression profiles of four immune cell subsets (CD4T, CD8T, B cells, and monocytes) simultaneously using transcriptome-wide RNA-Seq. The objective of this study was to determine which immune cell subsets contributed most prominently to the transcriptomic changes in whole blood from our previous study^15^. This study is the first to demonstrate an overlap as well as meaningful distinctions between different immune cell types implicated in differential gene expression in PTSD. Results revealed moderate correlations across the cell types for the 100 differentially expressed genes, with CD4T and CD8T being most similar to one another. Candidate gene analyses replicated earlier work identifying under-expression of *PF4* in monocytes as linked to PTSD. Gene expression differences in PTSD were largest and most distinct in monocytes. Monocytes are the most common type of leukocyte, and play a role in adaptive immunity^54^. Circulating blood monocytes are also precursors of macrophages. Activated macrophages and monocytes have been proposed to play a major role in pathogenesis of several central nervous system conditions and neurological disorders as well as PTSD^55,56^. Practically, these results suggest that future PTSD studies that wish to focus on gene expression in an isolated cell type might benefit from focusing on monocytes.

While our results replicated the upregulation in the candidate gene *PF4* reported by Neylan et al.^32^, the findings did not replicate the predominant downregulation of gene expression in monocytes in PTSD. The difference could be attributed to the specific threshold used in Neylan et al.^32^ for selecting candidate genes and the technical differences in assay systems. That is, we used RNA-Seq to establish the transcriptome profile, whereas Neylan et al. used microarrays. Furthermore, we considered a sequence of nominal *p*-value thresholds to ensure robustness of the results.

*FKBP5*, a gene that plays a role in the regulation of the glucocorticoid receptor and immunological responses to stress, was previously found to be upregulated in whole blood^15^. This study extended these results to show that the gene remained consistently upregulated in CD4T, CD8T, B cells, and monocytes, indicating that the effect of PTSD on *FKBP5* may not be cell specific. This study also identified another gene (*PI4KAP1*) that was consistently upregulated in CD4T (FDR < 0.05) and showed consistent trends across other cell types (nominal *p* < 0.05). Both *PI4KAP1* and *PI4KAP2* are two non-processed pseudogene partial copies of *PI4KA*, a lipid kinase and protein-coding gene. *PI4KAP2* was also consistently upregulated in all immune cell types, unsorted PBMC and whole blood (*p* < 0.05 in B cells and CD4T), whereas *PI4KA* was marginally upregulated in all cell types except monocytes in this study. These results suggest that the upregulations of *FKBP5* and *PI4KAP1* appear to be important targets for future study.

Prior studies have found a significant association between PTSD and onset of neurodegenerative diseases^3,4^. Several genes that were identified to be differentially expressed in the immune cell subsets have been implicated in neurodegenerative disorders. Despite the limited evidence regarding *PI4KAP1*, its parent gene, *PI4KA*, was implicated in late onset Alzheimer’s disease via its role in the synthesis of phosphatidylinositol phosphate^57–59^, which plays a diverse role in cell growth, differentiation and the nervous system^60,61^; whereas, the pseudogene *PI4KAP2* was found to be dysregulated in Huntington’s disease^62^. Both *PI4KAP1* and *PI4KAP2* are two non-processed pseudogene partial copy of PI4KA, and the presence of pseudogenes have been shown to complicate molecular genetic studies^63,64^. *Pi4KAP2* and *PI4KA* were also upregulated in all cell types (except *PI4KA* in monocytes), although it did not reach statistical significance. *REST* gene was significantly downregulated in monocytes comparing PTSD to control. *REST* gene is associated with neurodegeneration and has been identified as a master regulator of genes involved in neurogenesis and neuronal differentiation^65,66^. This gene was also found to be substantially reduced in individuals with mild cognitive impairment and almost absent in those with Alzheimer’s disease^67^. Another gene *SEPT4* identified to be significantly downregulated in monocytes, played a role in synaptic plasticity and neurodegeneration^68^, and has been found to be downregulated in the amygdala and the substantia nigra of patients with Parkinson’s disease^69^. This is particularly significant in our population where we recently demonstrated that our patients with PTSD have an increased risk of cognitive impairment^70^, worse cognitive dysfunction across domains linked with neurodegenerative diseases^71^, and reduced functioning in neurologically associated mobility conditions^72^.

The differentially expressed genes in PTSD identified within each cell type were enriched in gene sets related to immune response and inflammation. For example, gene sets in CD4T were involved in regulation of mast cells. Mast cells are the master regulators in immune response, and previous studies found that they may play an important role in innate and adaptive immune responses to inflammation and autoimmune diseases^73–76^. Mast cell activation in PTSD has been proposed to accelerate the pathogenesis of neurodegenerative diseases, including Alzheimer’s disease^77^. Furthermore, regulation of interferon-beta production emerged as the top gene sets in CD8T cells. Interferon-beta is involved in a wide range of biological activities in human immune system, including activating cytotoxic effector cells^78^, acting as antimicrobial agent^79^, and promoting maturation of leukocytes^80^. Our finding is in line with emerging evidence that interferon-beta is also implicated in several autoimmune diseases, including multiple sclerosis and Alzheimer’s disease^81–83^, whereas interferon signaling, particularly interferon-gamma has been shown to be associated with PTSD^84,85^. It is important to note that the pathway analyses were conducted on the top 100 genes ranked by the nominal *p*-values from differential expression analysis, where most of the genes did not reach statistical significance at FDR < 0.05. Thus, pending replication in independent samples, one should interpret the results from the pathway analyses with caution.

### Strengths and limitations

The current study had several strengths, including the first to profile gene expression in isolated immune cell types in PTSD using the cutting-edge RNA-Seq. Nonetheless, our findings must be considered in the context of several limitations. First, these data represent information from a cross-sectional study with a relatively small sample size selected based on our previous polygenic gene expression scores from whole-blood transcriptome study^15^, and thus we were not able to conclude whether immune cell subsets profiling is superior over whole blood. However, despite the small sample size, we were able to detect differentially expressed genes after adjusting for multiple comparisons for each cell type. The average percentage purity of the isolated immune cell subsets was >93% (Supplementary Materials), suggesting that the identified genes were likely not artefact of mixed signals arising from contamination of other cell types during sorting. Nevertheless, further work is needed that seeks to replicate these analyses in a larger sample. Second, this study only profiled gene expression in four types of immune cells, namely CD4T, CD8T, B cells, and monocytes, and identified distinct differential gene expression patterns comparing WTC-PTSD to non-PTSD. Future studies should investigate other blood cell type subsets including natural killer cells, neutrophils, and eosinophils. Furthermore, it is unclear to what extent results generalize to other traumatized samples and to females. Finally, some of the cell subsets exist in very small fractions, which may not yield sufficient number of cells for the bulk RNA-Seq technology. Future studies should examine these cell subsets using the single cell sequencing technology.

## Conclusion

The current study identified common and distinct gene signatures associated with PTSD in four subsets of leukocytes, indicating that the cell subpopulations may provide a valuable and to some degree independent source of information to refine the biomarker signature obtained from whole blood. In particular, monocytes showed the most differential gene expression in PTSD, suggesting that future work in isolated blood cells could focus on this subpopulation. Together with the results from pathway analysis, the gene expression profiles from cell subpopulations tentatively point to neurodegenerative mechanisms and to dysregulation in immune response. If independently replicated, these results add to a growing evidence suggesting that intervention strategies that target inflammatory responses may help to alleviate PTSD symptoms and related diseases.

## Supplementary information

Supplementary Table 6

Supplementary Methods

Supplementary Figure 1

Supplementary Figure 2

Supplementary Figure 3

Supplementary Figure 4

Supplementary Figure 5

Supplementary Figure 6

Supplementary Figure 7

Supplementary Figure 8

## Data Availability

The RNA-Seq data of the 39 samples will be available at the Gene Expression Omnibus (accession number GSE114407) upon publication.
