# Supplementary material for: Cell type-specific gene expression patterns associated with posttraumatic stress disorder in World Trade Center responders
Source: Transl Psychiatry. 2019 Jan 15;9:1. doi: 10.1038/s41398-018-0355-8 (PMC6341096; doi:10.1038/s41398-018-0355-8)
Supplement: Supplementary file 2 — Supplementary Methods [file 41398_2018_355_MOESM2_ESM.docx]

**Supplementary Methods**

*Flow sorts on frozen peripheral blood mononuclear cell (PBMC)*

Frozen PBMC were thawed, restored for 20 minutes in complete RPMI containing 10% FBS and 70 U/ml DNAse (Sigma), washed and counted. An aliquot of 30 million cells was resuspended in PBS containing 0.1% BSA (PBS/BSA), blocked with mouse IgG, and labeled for 30 minutes at room temperature with the following mouse anti-human antibodies: CD3-FITC (BD 340542), CD4-PE (BD 340670), CD8-BV510 (Biolegend 301048), CD14-APC-Cy7 (BD 641394), CD19-PE-Cy7 (Beckman Coulter IM3628U), and CD45-APC (Beckman Coulter IM2473U), as well as Live-dead Violet (ThermoFisher L34955). The cells were then washed in PBS/BSA, resuspended in 1 ml PBS/BSA, filtered through a 35 micron mesh, and diluted with 1 ml sort buffer (Hepes buffered saline with 1mM EDTA). Four-way sorting was performed on a BD FACSAria II sorter equipped with 488nm, 635nm, 405nm and 355nm (not used) lasers, using an 85-micron nozzle and 45-PSI pressure configuration. Sort selection criteria for all fractions included gating on single cells, CD45-positive and Live-dead negative events. Four fractions sorted simultaneously were monocytes, B-cells, CD4T, and CD8T cells. Fractions up to 1.9 million cells were collected into tubes containing 0.5 ml PBS/BSA. A sample of 1.5 million cells was taken prior to the staining procedure and compared to these fractions. Small samples were taken from the collected fractions for post-sorting purity analysis. Following completion of the sorting procedure all samples were centrifuged at 3500 rpm for 5 minutes, and most of the supernatant was removed. The pellets were transferred to an RNAse-free Eppendorf in about 300 μl buffer, and the tubes were rinsed once more with 300 μl PBS/BSA to transfer all cells. Cells were again pelleted at 2500 rpm for 5 minutes and most supernatant was removed from the pellet. Pellets were then snap-frozen in liquid nitrogen and stored until further processing at -80°C.

The processed PBMC samples did not contain more than 1% of cells outside of the boundaries of the CD45-positive mononuclear cell fraction, indicating that contamination of residual granulocytes from the initial gradient separation was minimal. Furthermore, the selection was made by stringent gating for CD14-high expressing cells only. Contamination with small granulocytes cannot be completely excluded but is expected to be minimal. T cell selection was performed by gating first on CD45-high CD3-high lymphocytes, and further separation by CD4-high and CD8-high populations, respectively, thereby excluding other mononuclear cells such as NK cells and DC.

The average percentages of purity of the sorted cell types were greater than 93%, i.e., CD4T (mean 96.2%, sd 1.54), CD8T (mean 94.3%, sd 2.56), monocytes (mean 93.8%, sd 2.17) and B-cells (mean 97.6%, sd 0.92) (Supplementary Figure 2A). In addition, as another validation of the percentage of purity from the cell sorting, we ran CIBERSORT^1^ on the each isolated immune cell subset RNA-Seq data. As shown in Supplementary Figure 2B, the estimated mean proportion of CD4T is 93.9% (sd 3.4) across the 39 CD4T RNA-Seq samples. The estimated proportion of other cell types, namely CD8T, monocytes, NK, B-cells, eosinophils (EOSIN) and neutrophils (PMNL) were all < 3%, supporting the high percentage of purity in isolated CD4T subtype. For B-cells RNA-Seq samples, the estimated proportion of B-cell is 88.1%, whereas the estimated proportion of other cell types were < 6% (see Supplementary Table 1 below, each cell reports the mean and sd over 39 samples). For the 39 CD8T RNA-Seq samples, the mean estimated proportion of CD8T was 39.1%. This could be attributed to the fact that it was harder to differentiate CD4T and CD8T using computational/bioinformatics tool such as CIBERSORT. This was also supported by the principal component plots which showed that CD4T and CD8T were most similar to each other (Figure 1 in main text and Supplementary Figure 4). The total estimated proportion of CD4T and CD8T was 87.3% in this case.

*Total RNA isolation from flow-sorted cells*

The purification of total and small RNA was prepared using the miRNeasy mini kit (Qiagen).  Two-million frozen flow-sorted cells were immediately lysed and resuspended with the addition of 700 μl of QIAzol Lysis Reagent. The lysate was then left at room temperature for 15 minutes to allow for adequate cell lysis. After addition of chloroform, the homogenate was separated into aqueous and organic phases by centrifugation. RNA partitioned to the upper, aqueous, phase while DNA partitioned to the interphase and proteins to the lower, organic, phase or to the interphase. The upper aqueous phase was extracted, and ethanol was added to provide appropriate binding conditions for all RNA molecules from 18 nucleotides upwards. The sample was then applied to the RNeasy Mini spin column, where the total RNA bound to the membrane and phenol and other contaminants were efficiently washed away.  On-column DNAse digestion was performed to remove residual genomic DNA contamination followed by additional washes. High quality RNA was then eluted in 60 μl of RNase-free water. Quantitative assessment of the purified total RNA was then accomplished using a Qubit Broad Range RNA kit (Thermofisher). The RNA was qualitatively evaluated by a 2100 Bioanalyzer (Agilent technologies).

*TruSeq Stranded Total RNA library preparation and sequencing*

The sequencing libraries were prepared with the TruSeq Stranded Total RNA kit (Illumina Inc), from 100ng total RNA.  After ribosomal depletion, the remaining RNA was purified, fragmented and primed for cDNA synthesis.  Fragmented RNA was then reverse-transcribed into first strand cDNA using random primers before then removing the RNA template and synthesizing a replacement strand, incorporating dUTP to generate ds cDNA.  AMPure XP beads (Beckman Coulter) were used to separate the ds cDNA from the second strand reaction mix resulting in blunt-ended cDNA.  A single ‘A’ nucleotide was then added to the 3’ ends of the blunt fragments.  Multiple indexing adapters containing a single ‘T’ nucleotide on the 3’ end of the adapter, were ligated to the ends of the ds cDNA thereby preparing them for hybridization onto a flow cell.   Adapter ligated libraries were amplified by PCR, purified using Ampure XP beads, and validated for appropriate size on a 4200 TapeStation D1000 Screentape (Agilent Technologies, Inc.).   DNA libraries were quantitated using KAPA Biosystems qPCR kit, and pooled together in an equimolar fashion, following experimental design criteria.  Each pool was denatured and diluted to 16pM for On-Board Cluster Generation and sequencing on a HiSeq2500 sequencer using a 100 cycle single-read cluster kit and rapid mode SBS reagents following the manufacturer’s recommended protocol (Illumina Inc.). The 39 samples were conducted in 3 processing batches, in which the distributions of PTSD and controls were approximately balance in the first two batches (Batch 1: 5 PTSD, 6 controls, Batch 2: 7 PTSD, 8 controls, Batch 3: 8 PTSD, 5 controls). In addition, the estimated surrogate variable from svaseq^2^ captured the processing batch differences (Supplementary Figure 2B).

*Comparison to the previously identified differentially expressed genes in whole blood*

We compared the differential expression analysis for PTSD status for each immune cell types to the previously identified 96 genes based on gene exon analysis of whole blood^3^ (Supplementary Table 6). 91/96 genes showed consistent sign in the estimated log2 fold change between the subset of 39 whole blood and the previous whole blood discovery cohort N=195. For other cell types, the proportions of genes with consistent sign in the estimated log2 fold change with the previous paper were 50/96 (CD4T), 57/96 (CD8T), 52/96 (B-Cells), 33/96 (monocyte) and 22/96 (unsorted PBMC). These results suggested that the differential expression in whole blood was partially attributed to differential gene expression in granulocytes which were removed during the separation of PBMC from whole blood.

*Weighted gene co-expression network analysis*

The weighted gene co-expression network analysis (WGCNA) ^4^ was used to identify modules of correlated genes. The WGCNA algorithm was performed on log transformed normalized gene body read counts. The Pearson correlation matrix was raised to the appropriate power (Supplementary Table 2) to achieve scale free topology in the discovery cohort. The minimum module size was set as 30, and the cut-offs for splitting and merging modules were 2 and 0.25, respectively. The number of modules identified within each cell type is provided in Supplementary Table 2. The gene expression profiles for each module were represented by the eigengene. The association between module eigengene and PTSD was performed using linear regression, adjusting for age and race, and significant modules were identified using FDR. The hub gene for each module, i.e., gene with the highest connectivity was reported. For each cell type, no module was significantly associated with PTSD at FDR < 0.05. The top 3 modules and the corresponding nominal p-values and hub genes were provided in Supplementary Table 3.

*Differential expression analysis comparing each pair of cell type within PTSD and control*

Differential expression analyses of RNA-Seq data generated from isolated CD4T, CD8T, B-cells and monocytes were performed using DESeq2^5^ software based on negative binomial generalized linear models, adjusting for age, race and the estimated surrogate variables to account for potential batch effects. Appropriate contrasts were setup to identify differentially expressed genes comparing each pair of cell type within PTSD and control, respectively. Differentially expressed genes comparing cell type A and B specific to PTSD group were defined as the genes with FDR < 0.05 in comparison of cell type A vs B within PTSD and p-values > 0.1 in comparison of cell type A vs B within control, and vice versa for genes specific to control group. Supplementary Table 4 displayed the number of differentially expressed genes for the pairwise cell type comparison which showed that there were substantial differences in gene expression between different cell types. CD4T and CD8T were relatively more similar as given by the smaller set of differentially expressed genes. The genes differentiating any two cell types within PTSD were approximately 3-5% of the total number of differentially expressed genes. Pathway analysis and gene ontology were carried out using the over representation analysis on the set of genes identified for each pairwise cell type comparison within PTSD and control group, respectively via the Bioconductor package clusterProfiler^6^. Only the gene sets from the comparison between CD4T vs B-cells and CD8T vs B-cells specific to PTSD yield over represented gene sets at FDR < 0.05. For other comparisons, the top 3 gene sets for each comparison were listed in Supplementary Table 5.

1. Newman AM, Liu CL, Green MR, Gentles AJ, Feng W, Xu Y *et al.* Robust enumeration of cell subsets from tissue expression profiles. *Nat Methods* 2015; **12**(5)**:** 453-457.

2. Leek JT. svaseq: removing batch effects and other unwanted noise from sequencing data. *Nucleic Acids Res* 2014; **42**(21).

3. Kuan PF, Waszczuk MA, Kotov R, Clouston S, Yang X, Singh PK *et al.* Gene expression associated with PTSD in World Trade Center responders: An RNA sequencing study. *Transl Psychiatry* 2017; **7**(12)**:** 1297.

4. Langfelder P, Horvath S. WGCNA: an R package for weighted correlation network analysis. *BMC Bioinformatics* 2008; **9:** 559.

5. Love MI, Huber W, Anders S. Moderated estimation of fold change and dispersion for RNA-seq data with DESeq2. *Genome biology* 2014; **15**(12)**:** 1.

6. Yu G, Wang L-G, Han Y, He Q-Y. clusterProfiler: an R package for comparing biological themes among gene clusters. *Omics: a journal of integrative biology* 2012; **16**(5)**:** 284-287.

**Supplementary Table 1:** Estimated cell proportions using CIBERSORT on RNA-Seq data of isolated immune cell subsets. EOSIN: eosinophils, PMNL: neutrophils. Each cell reports mean (sd) over 39 samples. Each column corresponds to the isolated immune cell samples. The rows correspond to the estimated cell proportions from CIBERSORT.

|  | CD4T RNA-Seq | CD8T RNA-Seq | B-cells RNA-Seq | Monocytes RNA-Seq |
| --- | --- | --- | --- | --- |
| CD4T % | 93.9 (3.4) | 48.2 (15.7) | 5.6 (2.3) | 3.5 (1.2) |
| CD8T % | 0 (0) | 39.1 (11.8) | 0 (0) | 0 (0) |
| B-cells % | 0.4 (0.7) | 0.3 (0.6) | 88.1 (2.9) | 0.5 (0.8) |
| Monocytes % | 1.6 (0.7) | 2.1 (1.0) | 0.3 (0.4) | 88.6 (3.4) |
| NK % | 2.9 (2.3) | 8.6 (5.6) | 2.0 (1.3) | 1.0 (0.7) |
| EOSIN % | 0.2 (0.3) | 0.4 (0.3) | 1.1 (0.7) | 1.0 (0.6) |
| PMNL % | 0 (0) | 0 (0) | 0 (0) | 0.03 (0.2) |

**Supplementary Table 2:** Estimated softpower and number of modules identified within each cell type.

| Cell Type | Estimated softpower | Number of modules identified |
| --- | --- | --- |
| CD4T | 4 | 23 |
| CD8T | 4 | 23 |
| B-cells | 5 | 20 |
| Monocytes | 6 | 29 |
| Whole blood | 7 | 18 |

**Supplementary Table 3:** The top 3 modules and the corresponding module sizes, nominal p-values and hub genes identified within each cell type.

|  | Size | p-value | Hub Gene |
| --- | --- | --- | --- |
| CD4T Module 1 | 161 | 0.039 | 161 |
| CD4T Module 2 | 358 | 0.052 | MAPK8IP3 |
| CD4T Module 3 | 206 | 0.106 | TMEM206 |
| CD8T Module 1 | 84 | 0.023 | SCRAP |
| CD8T Module 2 | 62 | 0.033 | MOB1A |
| CD8T Module 3 | 161 | 0.118 | SEPT7 |
| B-cells Module 1 | 43 | 0.214 | CLEC5A |
| B-cells Module 2 | 554 | 0.259 | ADAMTS5 |
| B-cells Module 3 | 43 | 0.273 | GTF2B |
| Monocytes Module 1 | 203 | 0.103 | FSD1 |
| Monocytes Module 2 | 445 | 0.108 | DAXX |
| Monocytes Module 3 | 439 | 0.150 | C10orf88 |

**Supplementary Table 4:** Number of significant genes comparing each pair of cell type at FDR < 0.05.

| Comparison | All | Unique to PTSD | Unique to control |
| --- | --- | --- | --- |
| CD4T vs CD8T | 6350 | 405 | 385 |
| CD4T vs B-cells | 11367 | 511 | 535 |
| CD4T vs Monocytes | 13777 | 383 | 364 |
| CD8T vs B-cells | 11438 | 465 | 496 |
| CD8T vs Monocytes | 13741 | 386 | 409 |
| B-cells vs Monocytes | 13660 | 411 | 452 |

**Supplementary Table 5:** Top 3 or list of gene set at FDR < 0.05 from the over representation analysis on differential expression genes specific to PTSD and control from each pairwise cell type comparison.

| Description | pvalue | FDR |
| --- | --- | --- |
| *CD4T vs CD8T specific to PTSD* |  |  |
| vacuole organization | 0.000428 | 0.562 |
| cellular modified amino acid biosynthetic process | 0.000688 | 0.562 |
| glycosphingolipid metabolic process | 0.000872 | 0.562 |
| *CD4T vs CD8T specific to control* |  |  |
| nucleoside bisphosphate biosynthetic process | 9.62E-05 | 0.0878 |
| ribonucleoside bisphosphate biosynthetic process | 9.62E-05 | 0.0878 |
| purine nucleoside bisphosphate biosynthetic process | 9.62E-05 | 0.0878 |
| *CD4T vs B-cells specific to PTSD* |  |  |
| SRP-dependent cotranslational protein targeting to membrane | 5.53E-06 | 0.0121 |
| cotranslational protein targeting to membrane | 9.28E-06 | 0.0121 |
| protein targeting to ER | 1.37E-05 | 0.0121 |
| establishment of protein localization to endoplasmic reticulum | 1.99E-05 | 0.0121 |
| ribosome biogenesis | 2.07E-05 | 0.0121 |
| protein localization to endoplasmic reticulum | 2.40E-05 | 0.0121 |
| ncRNA processing | 5.67E-05 | 0.0228 |
| chromatin remodeling at centromere | 6.04E-05 | 0.0228 |
| nuclear-transcribed mRNA catabolic process, nonsense-mediated decay | 6.90E-05 | 0.0231 |
| translational initiation | 8.32E-05 | 0.0251 |
| centromere complex assembly | 0.000172 | 0.0409 |
| DNA replication-independent nucleosome organization | 0.000172 | 0.0409 |
| rRNA processing | 0.000176 | 0.0409 |
| rRNA metabolic process | 0.000231 | 0.0497 |
| *CD4T vs B-cells specific to control* |  |  |
| cleavage involved in rRNA processing | 0.000119 | 0.358 |
| response to ionizing radiation | 0.000397 | 0.597 |
| response to alcohol | 0.000916 | 0.909 |
| *CD4T vs Mono specific to PTSD* |  |  |
| negative regulation of viral process | 0.000219 | 0.247 |
| regulation of symbiosis, encompassing mutualism through parasitism | 0.00037 | 0.247 |
| positive regulation of viral life cycle | 0.000529 | 0.247 |
| *CD4T vs Mono specific to control* |  |  |
| tRNA export from nucleus | 0.00113 | 0.869 |
| tRNA-containing ribonucleoprotein complex export from nucleus | 0.00113 | 0.869 |
| tRNA transport | 0.00142 | 0.869 |
| *CD8T vs B-cells specific to PTSD* |  |  |
| mitotic sister chromatid segregation | 1.56E-05 | 0.0477 |
| cellular respiration | 3.12E-05 | 0.0477 |
| sister chromatid segregation | 0.000128 | 0.13 |
| *CD8T vs B-cells specific to control* |  |  |
| tRNA metabolic process | 0.000227 | 0.65 |
| mitochondrial translational elongation | 0.00191 | 0.94 |
| mitochondrial translational termination | 0.00205 | 0.94 |
| *CD8T vs Mono specific to PTSD* |  |  |
| regulation of signal transduction by p53 class mediator | 0.000222 | 0.428 |
| protein polyubiquitination | 0.000446 | 0.428 |
| regulation of T cell migration | 0.000504 | 0.428 |
| *CD8T vs Mono specific to control* |  |  |
| mRNA transport | 0.000136 | 0.279 |
| mRNA export from nucleus | 0.000375 | 0.279 |
| mRNA-containing ribonucleoprotein complex export from nucleus | 0.000375 | 0.279 |
| *B-cells vs Mono specific to PTSD* |  |  |
| cellular response to alkaloid | 0.000427 | 0.755 |
| GTP metabolic process | 0.000939 | 0.755 |
| cofactor biosynthetic process | 0.00151 | 0.755 |
| *B-cells vs Mono specific to control* |  |  |
| nucleic acid transport | 0.000222 | 0.182 |
| RNA transport | 0.000222 | 0.182 |
| RNA localization | 0.000252 | 0.182 |

**Supplementary Table 6:** List of 96 genes identified from previous whole blood gene exon counts analysis ^3^. The log2 fold change and nominal p-value for each gene in each cell type are provided. Genes with nominal p-value < 0.05 are marked with asterisks. The cross-tabulations comparing the sign of the estimated log2 fold change from the previous whole blood and each cell type are provided at the end of each list.

**Supplementary Figure Legends**

**Supplementary Figure 1:** Overview of the cell specific gene expression analysis pipeline.

**Supplementary Figure 2:** A. Percentage of purity of the sorted immune cells. B. Comparison of the estimated surrogate variable from svaseq^2^ versus processing batches.

**Supplementary Figure 3:** Comparison of the estimated cell abundances obtained from different methods between PTSD and control. The p-values from two sample t-tests are printed above each cell type. A. Cell proportions estimated from cell sorting. B. Cell proportions estimated from CIBERSORT. C. Cell enrichment scores estimated from xCell. D. Pairwise correlation coefficients of the estimated cell abundances between methods across the 39 samples.

**Supplementary Figure 4:** Principal component analysis (PCA) of the normalized gene expression counts, left panel plots PC1 (x-axis) versus PC2 (y-axis), middle panel plots PC1 (x-axis) versus PC3 (y-axis) and right panel plots PC2 (x-axis) versus PC3 (y-axis). A. PCA of the top 1000 most variable genes across cell types. Each dot represented a sample, color coded by cell type (Red: B-cells, Sage: CD4T, Green: CD8T, Turquoise: monocytes, Sky blue: PBMC, purple: whole blood, plotting symbol +: control, solid circle: PTSD). B-E. PCA of the top 1000 most variable genes within each cell type, namely CD4T, CD8T, monocytes and B-Cells, respectively (Green solid circle: PTSD, red +: control)

**Supplementary Figure 5:** Volcano plots depicting transcriptome-wide differential gene expression patterns associated with PTSD in CD4T (A), CD8T (B), B-cells (C) and monocytes (D). Each dot represented a gene, red dots corresponded to the genes differentially expressed at FDR < 0.05. x-axis denoted the estimated log2 fold change, y-axis denoted the negative log of unadjusted p-values from differential expression analysis.

**Supplementary Figure 6:** Proportion of downregulated genes among the differentially expressed genes in PTSD (y-axis) at different nominal p-value thresholds (x-axis) for each cell type.

**Supplementary Figure 7:** Heatmap of the 34 differentially expressed genes at FDR < 0.05 from the joint analysis of all four immune cell types. The number printed in each heatmap cell corresponded to the estimated unadjusted p-value from DESeq2, and the color corresponded to the magnitude of the estimated log2 fold change (blue: down-regulated in PTSD, red: up-regulated in PTSD).

**Supplementary Figure 8:** Pairwise correlation coefficients comparing the effect of cell type adjustment (cell sorting versus CIBERSORT versus xCell) of the estimated log2 fold change and the negative log p-value transcriptome-wide from the differential expression analysis comparing cases (PTSD) to control for both PBMC and whole blood analysis.
