# Supplementary figures and images for: Cell type-specific gene expression patterns associated with posttraumatic stress disorder in World Trade Center responders
Source: Transl Psychiatry. 2019 Jan 15;9:1. doi: 10.1038/s41398-018-0355-8 (PMC6341096; doi:10.1038/s41398-018-0355-8)

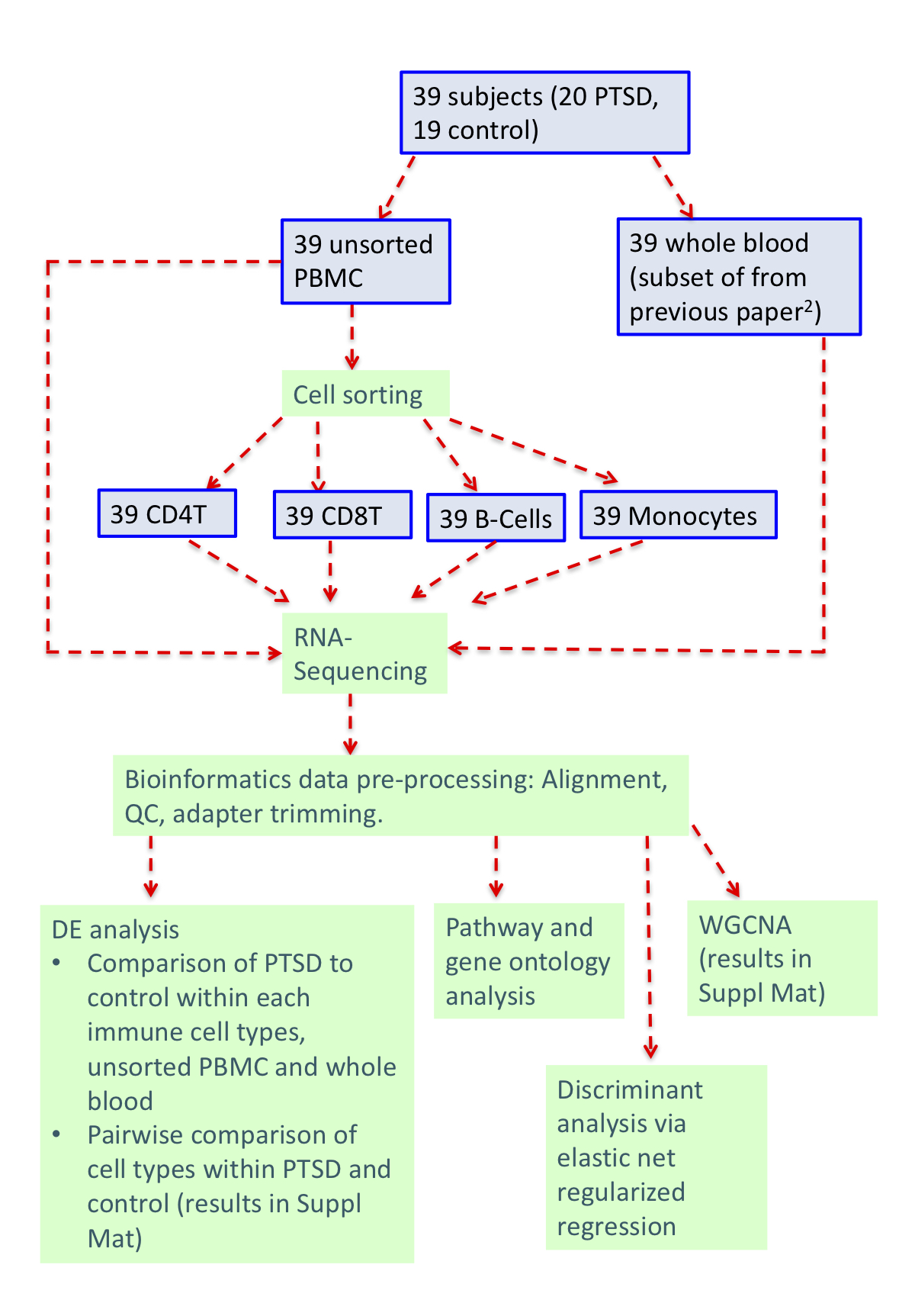

Supplement: Supplementary file 3 — Supplementary Figure 1 [file 41398_2018_355_MOESM3_ESM.jpg]

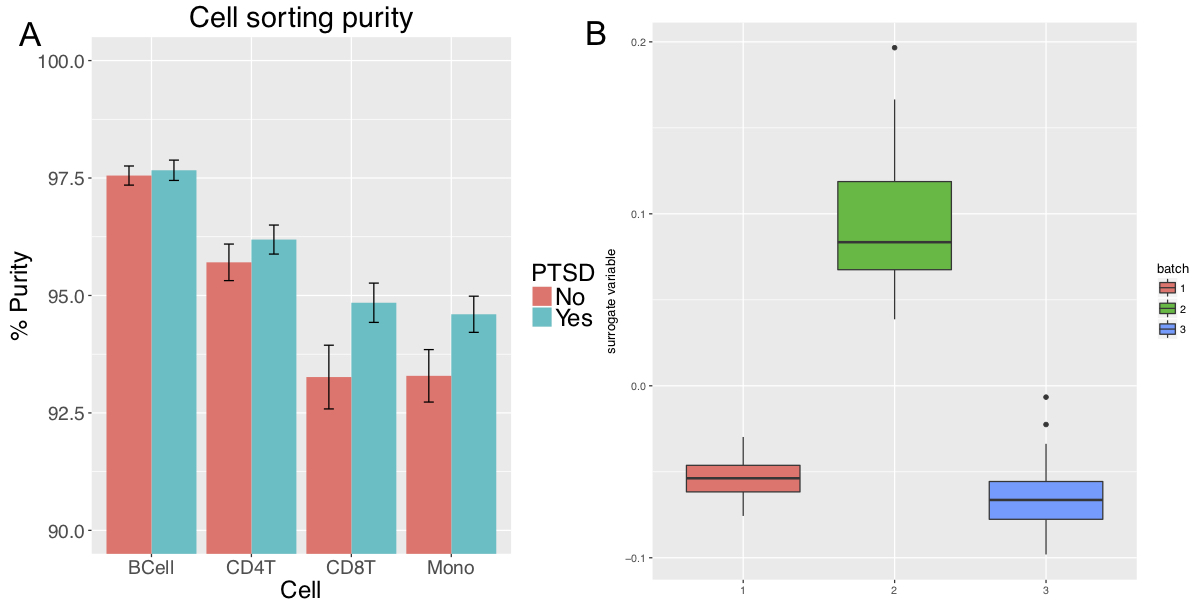

Supplement: Supplementary file 4 — Supplementary Figure 2 [file 41398_2018_355_MOESM4_ESM.jpg]

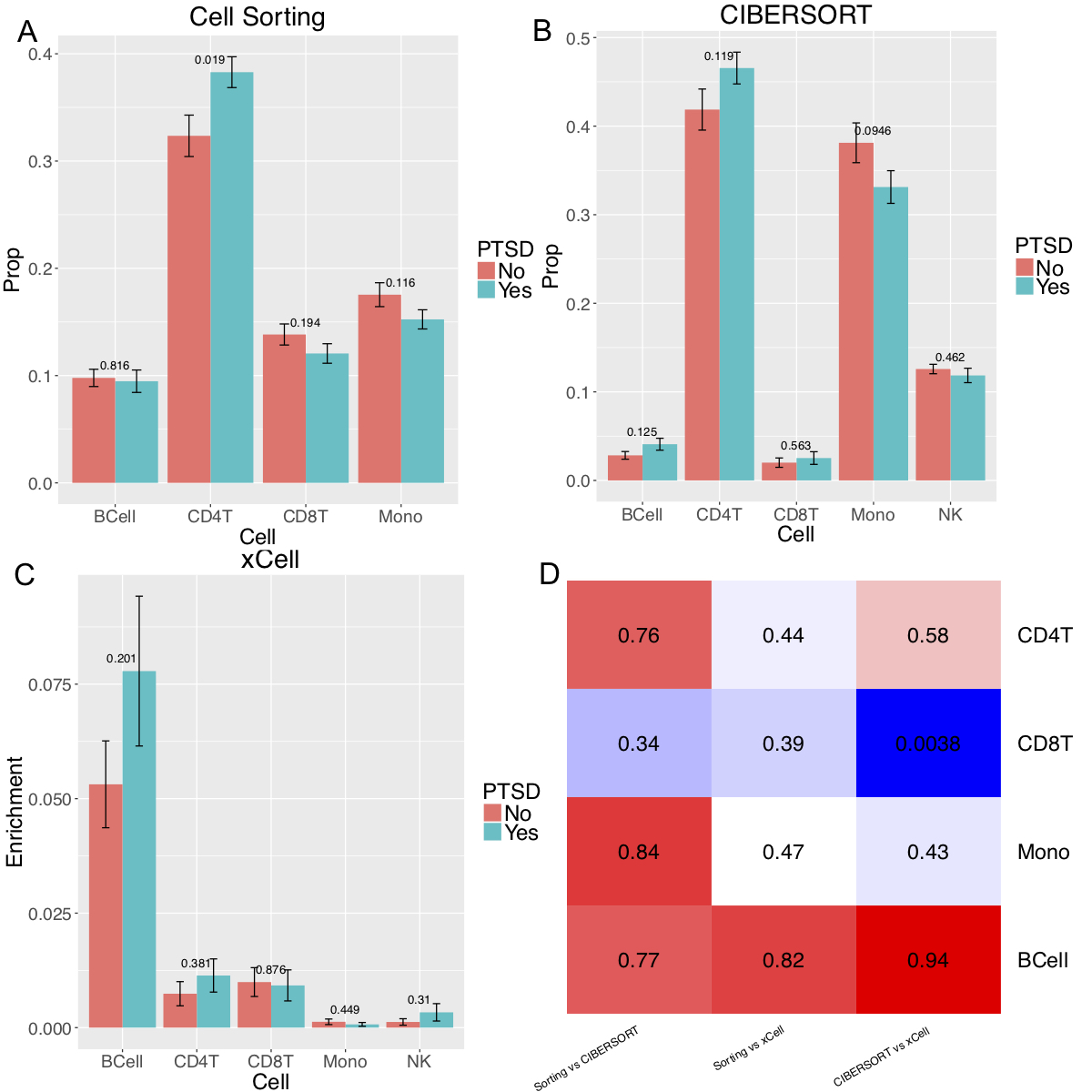

Supplement: Supplementary file 5 — Supplementary Figure 3 [file 41398_2018_355_MOESM5_ESM.jpg]

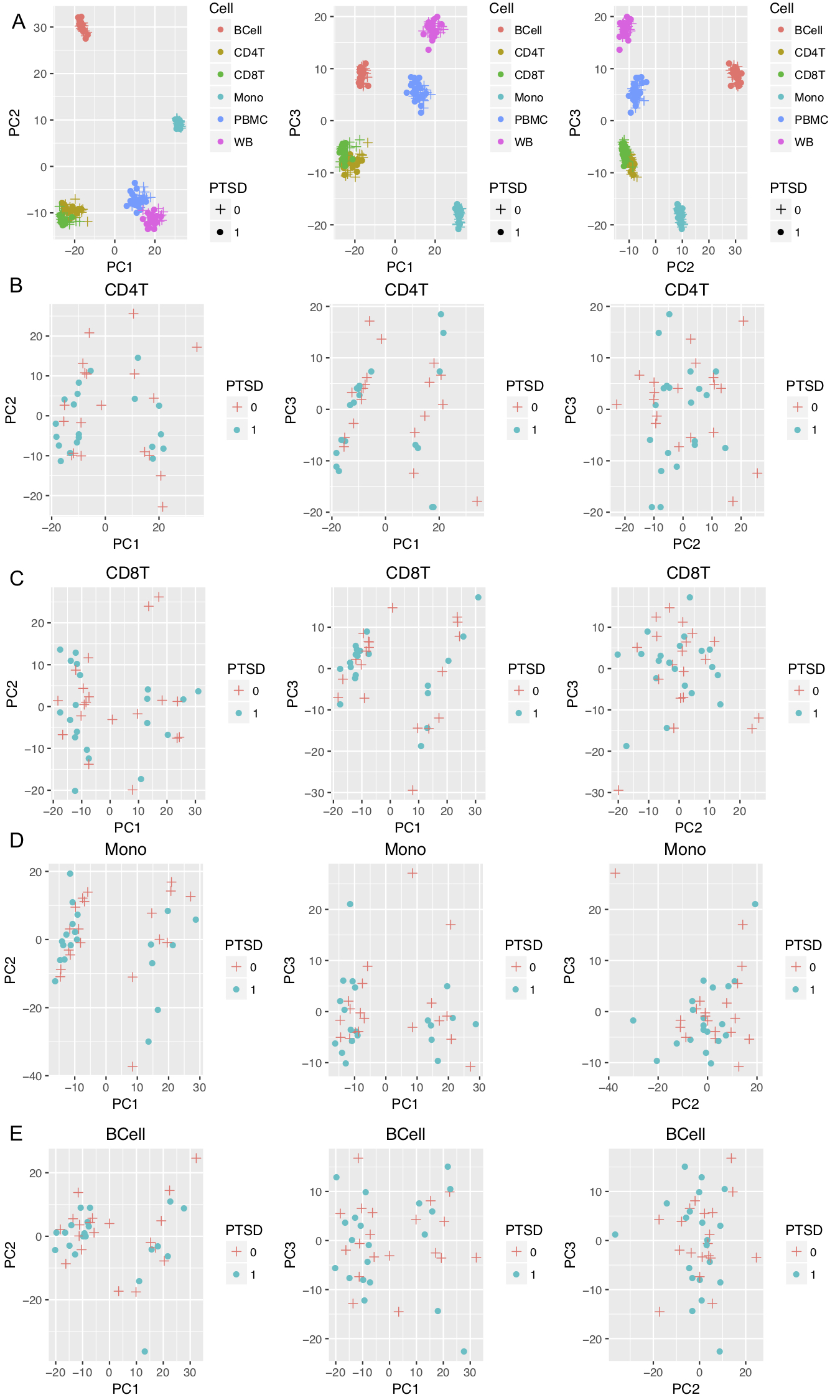

Supplement: Supplementary file 6 — Supplementary Figure 4 [file 41398_2018_355_MOESM6_ESM.jpg]

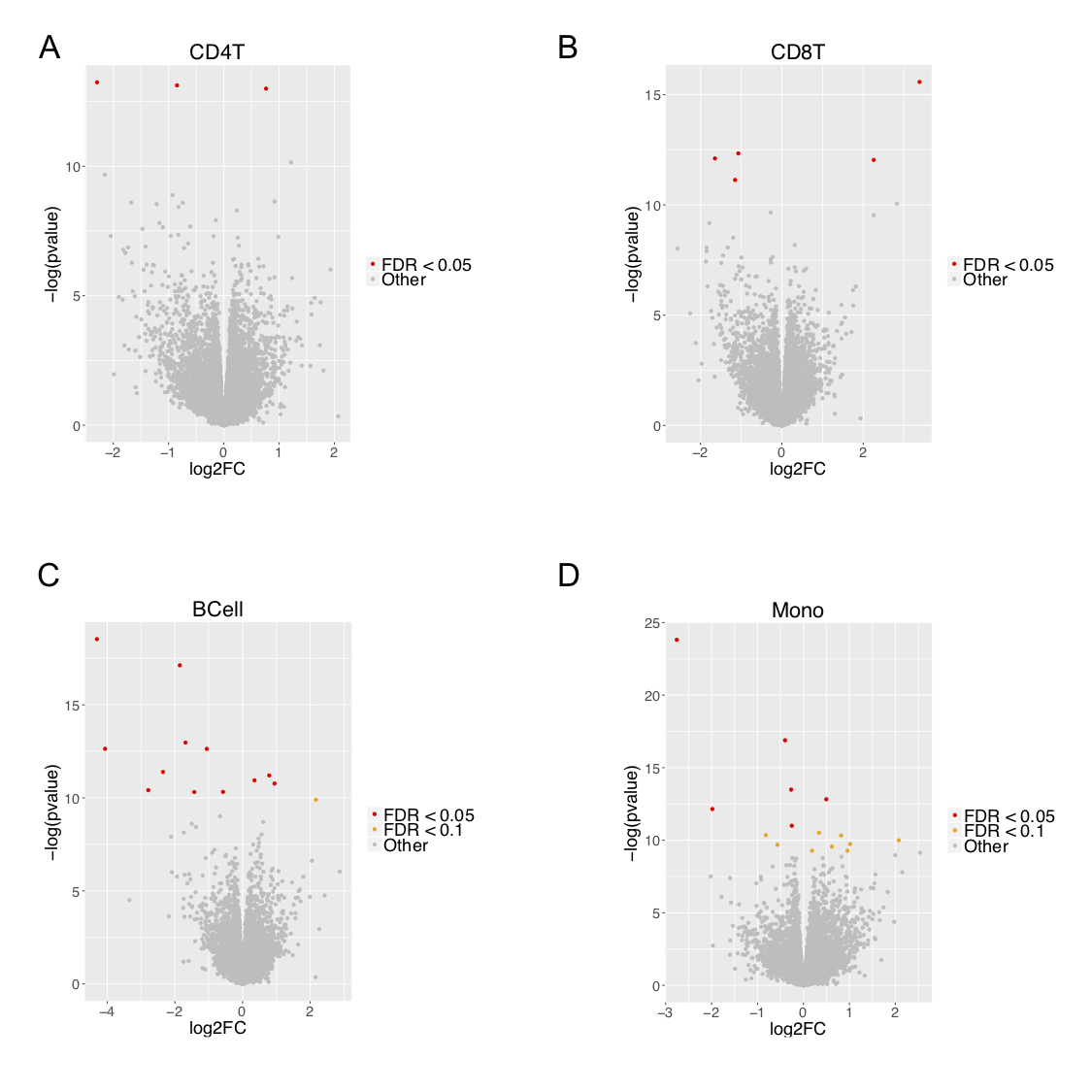

Supplement: Supplementary file 7 — Supplementary Figure 5 [file 41398_2018_355_MOESM7_ESM.jpg]

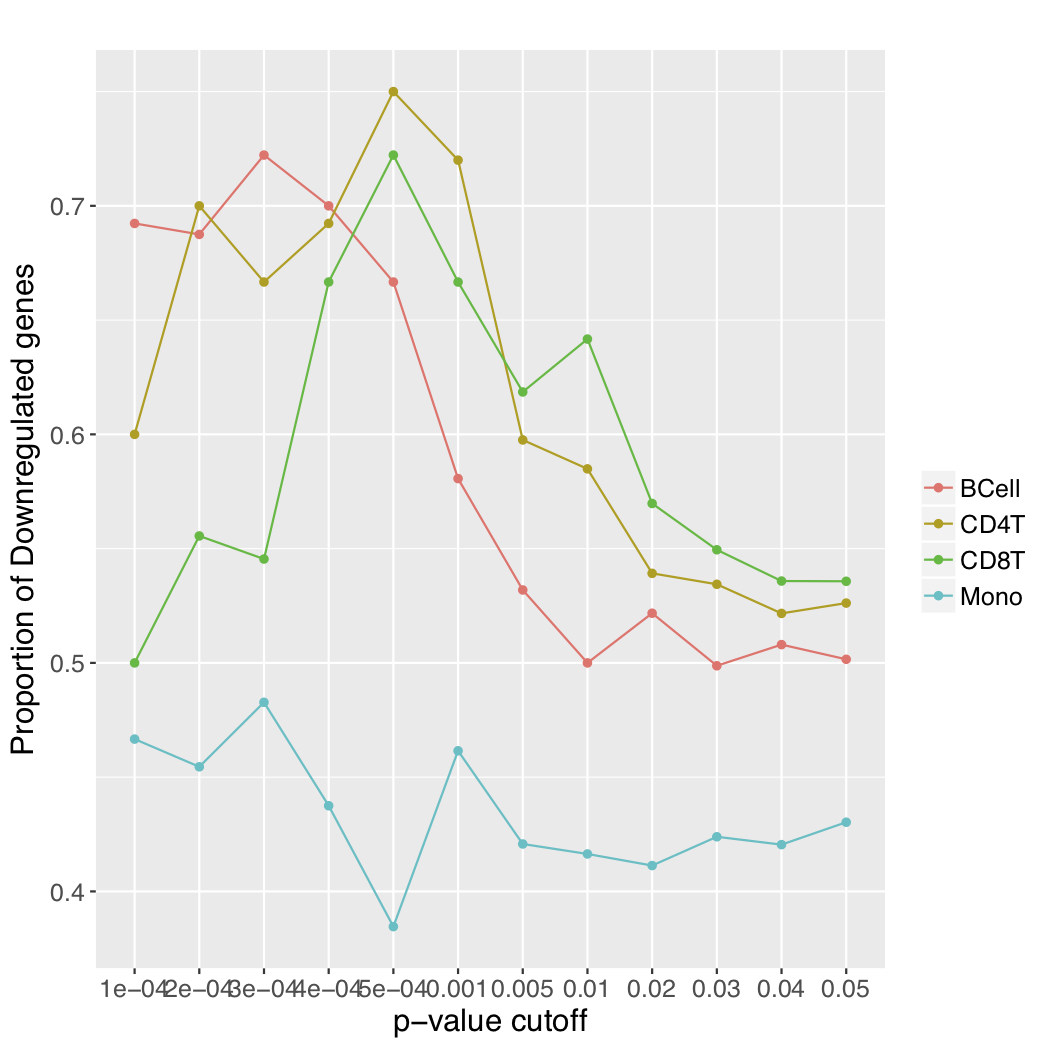

Supplement: Supplementary file 8 — Supplementary Figure 6 [file 41398_2018_355_MOESM8_ESM.jpg]

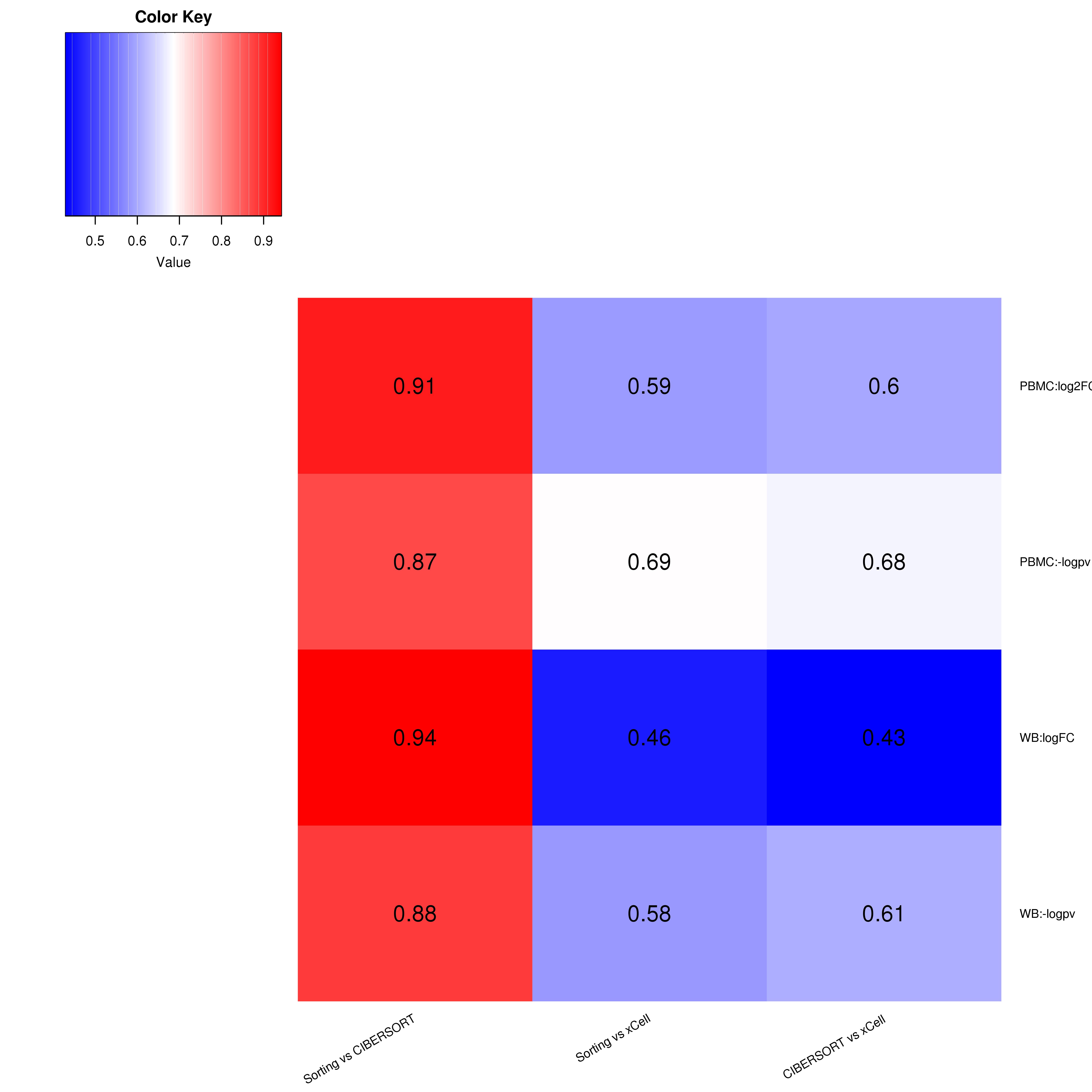

Supplement: Supplementary file 10 — Supplementary Figure 8 [file 41398_2018_355_MOESM10_ESM.jpg]
